# Sitosterolemia With Atherosclerosis in a Child: A Case Report

**DOI:** 10.3389/fped.2021.668316

**Published:** 2021-06-11

**Authors:** Hongjun Ba, Huimin Peng, Xiufang He, Liangping Cheng, Yuese Lin, Xuandi Li, Huishen Wang, Youzhen Qin

**Affiliations:** ^1^Department of Pediatric Cardiology, Heart Center, The First Affiliated Hospital, Sun Yat-sen University, Guangzhou, China; ^2^Key Laboratory on Assisted Circulation, Ministry of Health, Guangzhou, China

**Keywords:** sitosterolemia, atherosclerosis, hypercholesterolemia, xanthoma, genetic, mutation

## Abstract

**Introduction:** Sitosterolemia is a rare condition in children and is often misdiagnosed as familial hypercholesterolemia. Serious complications can result if not treated promptly and effectively. When pediatric patients are diagnosed with sitosterolemia, vascular, and cardiac studies are important to evaluate for the presence of atherosclerosis. Few cases of severe atherosclerotic heart disease in children with sitosterolemia have been reported, making this case worthy of presentation.

**Case Presentation:** Here, we report a case of sitosterolemia in an 8-year-old child. The patient presented with severe hypercholesterolemia and xanthoma. He was diagnosed two and a half years prior with familial hypercholesterolemia because his father had elevated cholesterol levels. After conventional treatment, the patient was dissatisfied with lipid level control and visited our hospital for further management. Genetic tests of the patient and parents found mutations in intron 7 (NM 022436.2, c.904+1G>A) and intron 9 (NM 022436.2, C. 1324+1de1G) of ABCG5. The 7 intron mutation was from his mother, and the 9 intron mutation was from his father. The patient was diagnosed with sitosterolemia.

**Results:** The child was treated with ezetimibe, a low plant sterol diet, and clopidogrel anticoagulant therapy. After 3 months of treatment, the blood lipid level was significantly lower.

**Conclusion:** Genetic testing should be completed as soon as possible to avoid misdiagnosis in children with abnormally elevated hypercholesterolemia who have a family history of elevated cholesterol. In addition, clinicians should rule out great arterial lesions and be vigilant in evaluating patients for systemic arterial disease and atherosclerosis.

## Introduction

Sitosterolemia, also known as phytosteremia, is a rare autosomal recessive genetic disease (OMIM210250) that causes dysmetabolism of plant sterols (1). Mutations in the ABCG5 or ABCG8 gene lead to a loss of function of the ATP-binding cassette. ABCG5 and ABCG8 both encode sterolin 1 and 2 proteins expressed in the small intestine and liver cells, and actively transport plant sterols to the human intestine and bile ducts (2, 3). These transporters have also been found in hepatocytes, where they secrete cholesterol and plant sterols from liver cells into the bile (4). Thus, the mechanism of sitosteremia is defective transport of these sterols from the intestinal epithelium back into the lumen and from the liver into the bile. Sitosterolemia is associated with skin xanthoma, atherosclerosis, arthritis, liver and splenomegaly, lipid-type red blood cells, large platelets, and thrombocytopenia (5, 6). Most patients with sitosterolemia show significant increases in total blood cholesterol and low-density lipoprotein cholesterol. Coronary atherosclerosis is more common in adults; pathological vascular damage is rare in children. Therefore, we share our experience in the diagnosis and treatment of sitosterolemia in a child caused by a double heterozygous mutation in the ACBG5 gene.

## Case Presentation

### Patient Information

An eight-and-a-half-year-old boy was admitted to our hospital for hyperlipidemia. Two and a half years ago, the child was brought to a local hospital with multiple red papules scattered on the elbows, knees, and buttocks. Laboratory tests revealed severely elevated blood lipids and a total cholesterol level of 15.13 mmol/L. A lumpectomy was performed. Pathological examination revealed a xanthoma. Since the father had hyperlipidemia, the patient was misdiagnosed with familial hypercholesterolemia and treated with a low-fat diet and oral administration of levocarnitine. After treatment, his lipid level went down a little bit, but remained high.

### Clinical Findings

**Figure 1:**
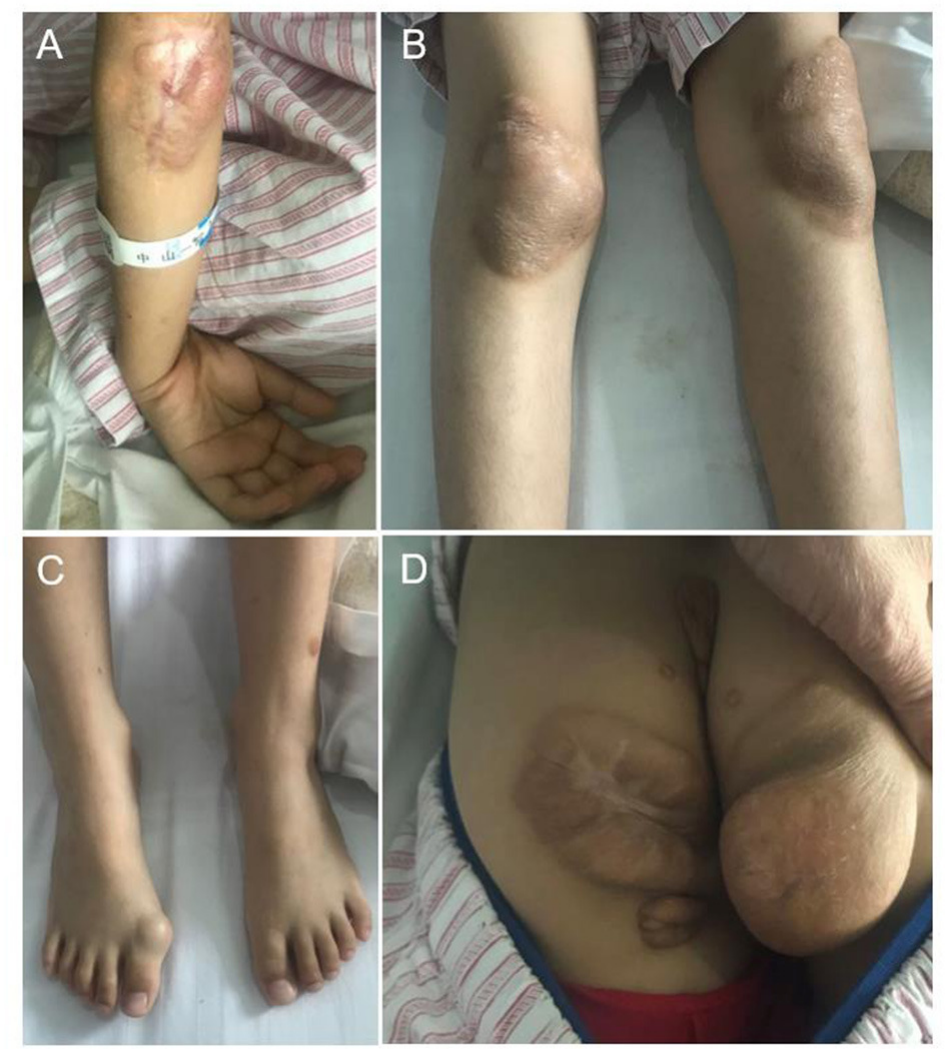
Cutaneous features of the case. **(A)** Xanthoma on the elbow. **(B)** Xanthoma on both knees. **(C)** Xanthoma on the great toe joint. **(D)** Xanthoma on the buttocks.

Physical examination on admission showed that the weight and height of the patient was lower than those of the same age group, with a weight of 19.6 kg (−2.2SD) and height of 116 cm (−2.2SD). Blood pressure was normal in all extremities. Several nodules were scattered on both upper eyelids, elbows, right fingertips, buttocks, knees, double popliteus, heel, and on the right big toe (Figure 1). The largest xanthoma, ~6.0 cm × 6.0 cm × 6.0 cm, was located on the right buttock. There was no yellowing, rash, or bleeding point in the skin, and no enlargement of the superficial lymph nodes. There were no abnormalities in the heart, lung, or abdomen. No swelling of the limbs was observed.

Laboratory tests revealed a total cholesterol of 7.3 mmol/L, triglyceride 0.80 mmol/L, high-density cholesterol (HDL-C) 0.82 mmol/L, and low-density cholesterol (LDL-C) 5.70 mmol/L. No abnormalities were found in liver and kidney function, blood glucose, insulin, Epstein Barr (EB) virus serum antibodies, and tuberculosis antibody. The growth hormone stimulation test results were negative. Vasculitis index and serological antibody of systemic lupus erythematosus (SLE) were also negative. Erythrocyte sedimentation rate was 81 mm/h, C-reactive protein (CRP) 41.78 mg/L, and interleukin-6 23.26 pg/mL. There was no problems with either coagulation or blood routine tests.

The patient's 5-year-old sister was also tested for blood lipids and had elevated LDL cholesterol. The patient's sister also had xanthoma on both elbows. The patient's mother had normal lipid levels.

### Diagnostic Assessment

**Figure 2:**
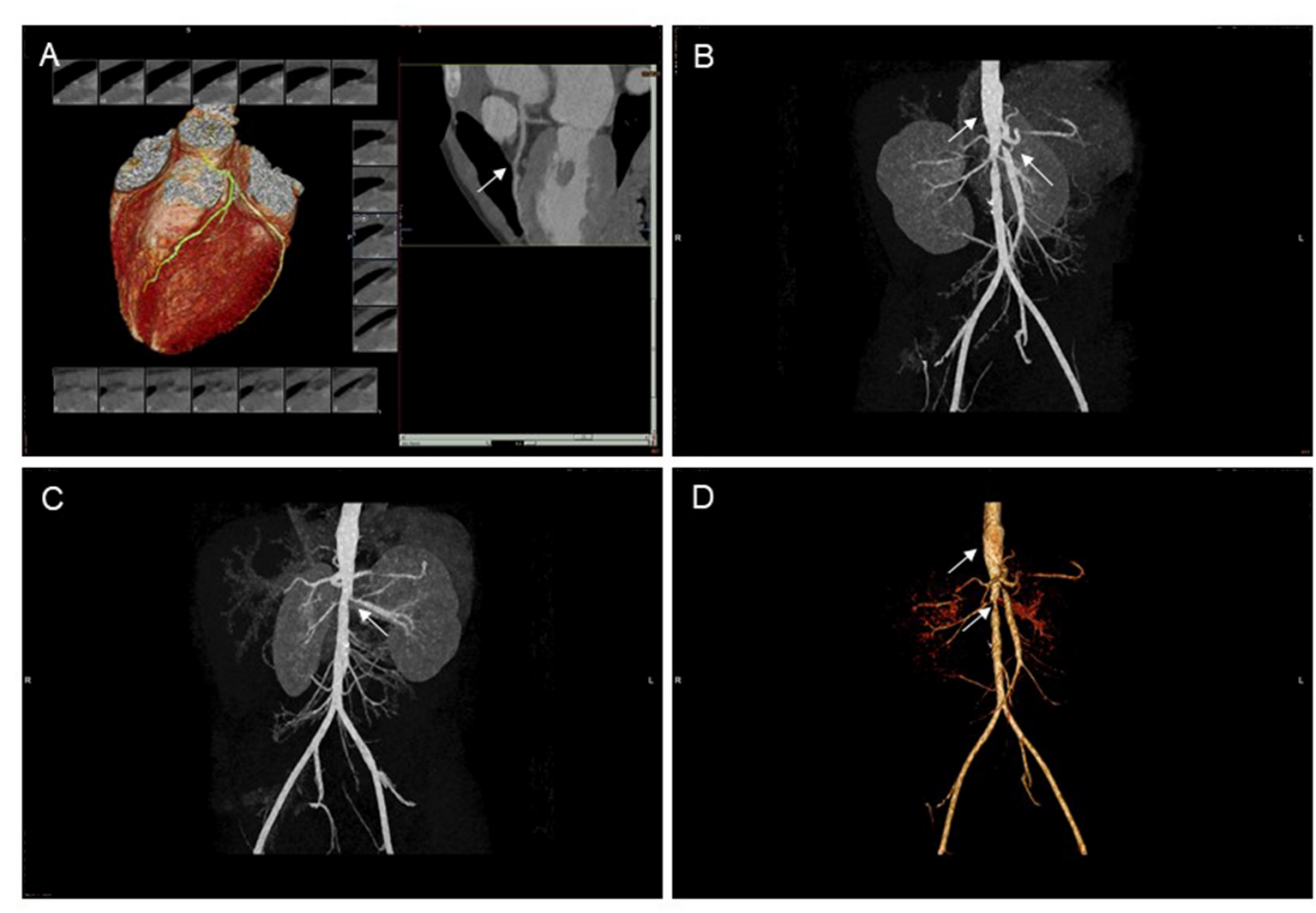
CT scan of coronary arteries and abdominal vessels. **(A)** Coronary artery stiffness, left coronary artery segmental stenosis. **(B–D)** The abdominal aortic wall is thickened and unevenly dilated, and the opening of the proximal bilateral renal artery, the celiac trunk and the superior mesenteric artery are narrowed. CT, computed tomography.

Dynamic electrocardiogram (ECG) indicated frequent ventricular premature beats, paroxysmal supraventricular tachycardia, and occasional ventricular premature beats. Coronary CTA showed thickening of the aortic wall, stiffness of the coronary arteries, and changes in left and right arteries segmental stenoses (Figure 2A).

Abdominal computed tomography angiography (CTA) showed a thickened abdominal aortic wall, unevenly dilated, bilateral proximal renal artery, celiac trunk, and narrowing of the superior mesenteric artery (Figures 2B–D).

**Figure 3:**
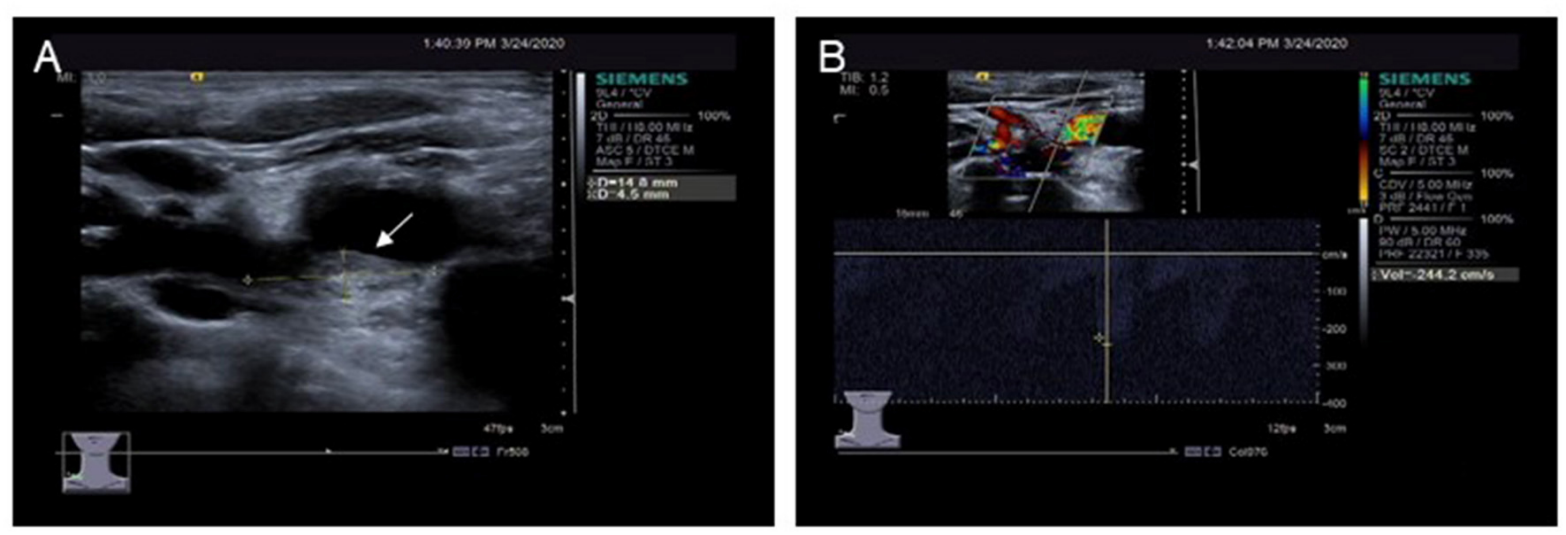
Ultrasound examination of the right clavicular artery. **(A)** A plaque approximately 4.5 mm × 14.8 mm in size is seen in the initial segment of the right clavicular artery. **(B)** Blood flow speeds up in the plaques of the right clavicular artery.

Cervical vascular ultrasound showed initial stenosis of the right subclavian artery (jugular stenosis <50%) with partial blood theft and bilateral jugular stenosis <50% (Figure 3). Carotid artery ultrasound showed no abnormality.

No abnormalities were found on magnetic resonance imaging (MRI) and magnetic resonance angiography (MRA) of the brain.

Genetic testing was performed by NGS (next-generation sequencing) at the Guangzhou KingMed Center for Clinical Laboratory (Guangzhou KingMed Diagnostics Group Co., Ltd). DNA samples from the patient and his parents were collected with their consent. Detection of gene loci include: LDLR (LDL receptor), APOB (apoB), STAP1 (signal-transducing adaptor protein), PCSK9 (proprotein convertase subtilisin/kexin type9), LDLRAP1 (LDL receptor adaptor protein 1), CYP27A1 (sterol 27 hydroxylase), APOE (apoE), ABCG5, ABCG8, and LIPA (lysosomal acid lipase A). The Sanger method was used to verify the results. Genetic tests of the patient and the parents showed mutations; the patient had splicing mutations in intron 7 (NM 022436.2, c.904+1G>A) and intron 9 (NM 022436.2, c.1324+1de1G) of ABCG5. The Human Splicing Finder (HSF, http://www.umd.be/HSF3/HSF.html) showed both mutations are pathogenic. The 7 intron mutations were from his mother, and the 9 intron mutations were from his father. His sister was not tested (Table 1).

**Table 1:** Serum LDL-cholesterol level, gene mutation and clinical manifestation of the family.

			**Genetic mutations**				
	**Age/gender**	**LDL-cholesterol (mmol/L)**	**Gene**	**Location**	**cDNA levels**	HSF^*^	**Clinical presentation**
Index case	8y/M	15.3	ACBG5	Intron7 Intron9	c.904+1G>A c.1324+1delG	Pathogenic	Xanthoma
Father	37y/F	8.0	ACBG5	Intron9	c.1324+1delG	Pathogenic	–
Mather	35y/F	2.1	ACBG5	Intron7	c.904+1G>A	Pathogenic	–
Sister	5y/F	5.2	Didn't do genetic testing			–	–

* *HSF is the abbreviation of Human Splicing Finder, which is a functional prediction index of mutant genes*.

Based on the presence of xanthoma, hyperlipidemia, vascular damage, and genetic results, the diagnosis was sitosterolemia and lipid-related arterial disease.

### Therapeutic Intervention

After definite diagnosis, the patient was administered ezetimibe (5 mg qd) and clopidogrel anticoagulation (25 mg qd) therapy. A low plant sterol diet and an increase in appropriate exercise were advised.

### Follow-Up and Outcomes

Regular follow-up in pediatric clinics, every 4–6 weeks, was recommended. After 2 months of treatment, the blood lipid level was decreased, total cholesterol was 4.3 mmol/L, low-density lipoprotein cholesterol was 2.5 mmol/L, triglyceride was 1.5 mmol/L, and high-density lipoprotein cholesterol was 1.02 mmol/L. Xanthoma of the extremities became soft. Imaging examination of vascular lesions has not been reviewed.

## Discussion

Sitosterolemia is a rare disorder of lipid metabolism. It was first reported by Bhattacharyya and Connor (7). Studies have shown that sitosterolemia is caused by mutations in the gene ABCG5 or ABCG8, which encodes the sterolin protein that has the function of transporting plant sterols (2–4). Patients with sitosterolemia have high cholesterol levels, which can easily be misdiagnosed as familial hypercholesterolemia, leading to inappropriate treatment. The patient we reported had a significant increase in cholesterol and xanthoma 2.5 years ago. He was misdiagnosed with familial hypercholesterolemia and not given targeted treatment. Hypercholesterolemia and xanthoma are two of the most common manifestations in patients with sitosterolemia (8). Our patient had cholesterol levels up to 15 mmol/L. According to the literature, about 85% of patients with sitosterolemia have xanthoma (9); therefore, patients with xanthoma should be evaluated to rule out sitosterolemia.

At present, the diagnosis of sitosterolemia mainly depends on the level of plant sterols and gene sequencing. Plant sterols cannot be distinguished from cholesterol by routine methods; this process can only be done by gas chromatography-mass spectrometry (GC-MS) (10, 11). General medical institutions do not have this form of technology, making it easy to misdiagnose sitosterolemia as hypercholesterolemia. Gene sequencing is the primary diagnostic tool and is more readily accessible than GC-MS. Mutations in ABCG5 and ABCG8 genes have been found to be the main cause of the disease (12). Mutations in ABCG5 were more than twice as frequent as those in ABCG8 in reported patients with sitosterolemia. ABCG5 gene mutation sites are mainly exon mutations with a small number of patients exhibiting intron mutations. Our patient had intron 7 and intron 9 mutations. The father had a mutation in intron 9 of the ABCG5 gene which manifested as hypercholesterolemia. The mother had a mutation of intron 7 of the ABCG5 gene and had normal lipids. The two intron mutations in our patient with severe hyperlipidemia suggested different introns' pathogenicity. In addition, due to family factors, the patient's sister was not tested for the gene. However, considering the presence of hypercholesterolemia and xanthoma, it could be inferred that she was likely to have the mutant gene.

Our patient had a high cholesterol level, which was consistent with the literature (13, 14). Brinton et al. (14) found there is an association between marked hypercholesterolemia and sitosterolemia, and the diagnosis of sitosterolemia should be considered in subjects with LDL-C levels ≥190 mg/dL. Patients with sitosteremia have high cholesterol levels due to defective transport of these sterols from the intestinal epithelial ileum and from the liver to the bile. In addition to high cholesterol levels, sitosteremia patients have other symptoms, such as xanthoma, atherosclerosis, arthritis, liver and splenomegaly, lipid-type red blood cells, but the phenotypes of each patient varied greatly. Coronary atherosclerosis may be the main cause of sudden death in patients with sitosterolemia. Several cases of sitosteremia were associated with significant premature coronary heart disease, many before the age of 20 years, and in some cases causing sudden death (15–17). Therefore, attention should be paid to the cardiovascular complication of sitosterolemia.

Because atherosclerosis is rare in children, atherosclerosis in pediatric patients with sitosterolemia is easily misdiagnosed as arteritis, especially when it is accompanied by elevated inflammatory markers. Our patient was examined for connective tissue disease, arteritis, tuberculosis, and other diseases which may manifest as atherosclerosis, and no abnormalities were found. Therefore, instead of supporting the diagnosis of vasculitis, vascular ultrasound showed atherosclerotic plaques in the right clavicular artery, further confirming the presence of sitosterolemia-associated atherosclerosis. To our knowledge, there have been no reports of multiple arterial lesions in children with sitosterolemia; only carotid artery plaques have been reported (18). Another important manifestation of our patient was frequent premature ventricular beats. There were no clinical symptoms or ECG ischemia. The premature beats may have been related to coronary artery stenosis; therefore, long-term follow-up is necessary.

Dietary therapy is the main treatment of sitosterolemia (19). Most of the reported patients were treated with strict restriction of plant sterols and partial restriction of animal sterols. As a result, xanthoma subsided and total cholesterol was reduced to normal; however, plant sterols remained high and drug treatment was often required. Ezetimibe is an effective drug for controlling elevated cholesterol (19, 20). Our patient was treated with diet and ezetimibe, and the lipid levels were significantly lower than before treatment. It is not known whether atherosclerosis decreases with a decrease in cholesterol levels, and further observation is needed. In addition, patients with coronary artery involvement may require anticoagulant therapy to prevent coronary embolism.

In conclusion, doctors should be vigilant against sitosterolemia, avoid misdiagnosis, and pay attention to the evaluation of complications, especially atherosclerosis, when treating children with severe hypercholesterolemia.

## Data Availability Statement

The raw data supporting the conclusions of this article will be made available by the authors, without undue reservation.

## Ethics Statement

This case report was approved by the Ethics Committee of the First Affiliated Hospital of Sun Yat-sen University. Written informed consent was obtained from patients' parents for publication of this case report and any potentially identifying information.

## Author Contributions

All authors listed have made a substantial, direct and intellectual contribution to the work, and approved it for publication.

## Conflict of Interest

The authors declare that the research was conducted in the absence of any commercial or financial relationships that could be construed as a potential conflict of interest.
